# Supplementary material for: Inflammation status modulates the effect of host genetic variation on intestinal gene expression in inflammatory bowel disease
Source: Nat Commun. 2021 Feb 18;12:1122. doi: 10.1038/s41467-021-21458-z (PMC7892863; doi:10.1038/s41467-021-21458-z)
Supplement: Supplementary file 1 — Supplementary Information [file 41467_2021_21458_MOESM1_ESM.pdf]

Supplementary Information

Inflammation status modulates the effect of host genetic variation on intestinal gene expression in inflammatory bowel disease

Supplementary figure1. Cell type-enrichment differs between inflamed and non-inflamed tissues. X axis indicates cell type-enrichment scores derived from xCell. Y axis indicates 28 cell types present in intestinal mucosa. *significant difference (two-sided Wilcoxon test, FDR <0.05) Box plots show medians and the first and third quartiles (the 25th and 75th percentiles), respectively. The upper and lower whiskers extend the largest and smallest value no further than 1.5*IQR (n =280 samples, source data are provided as a Source Data file).

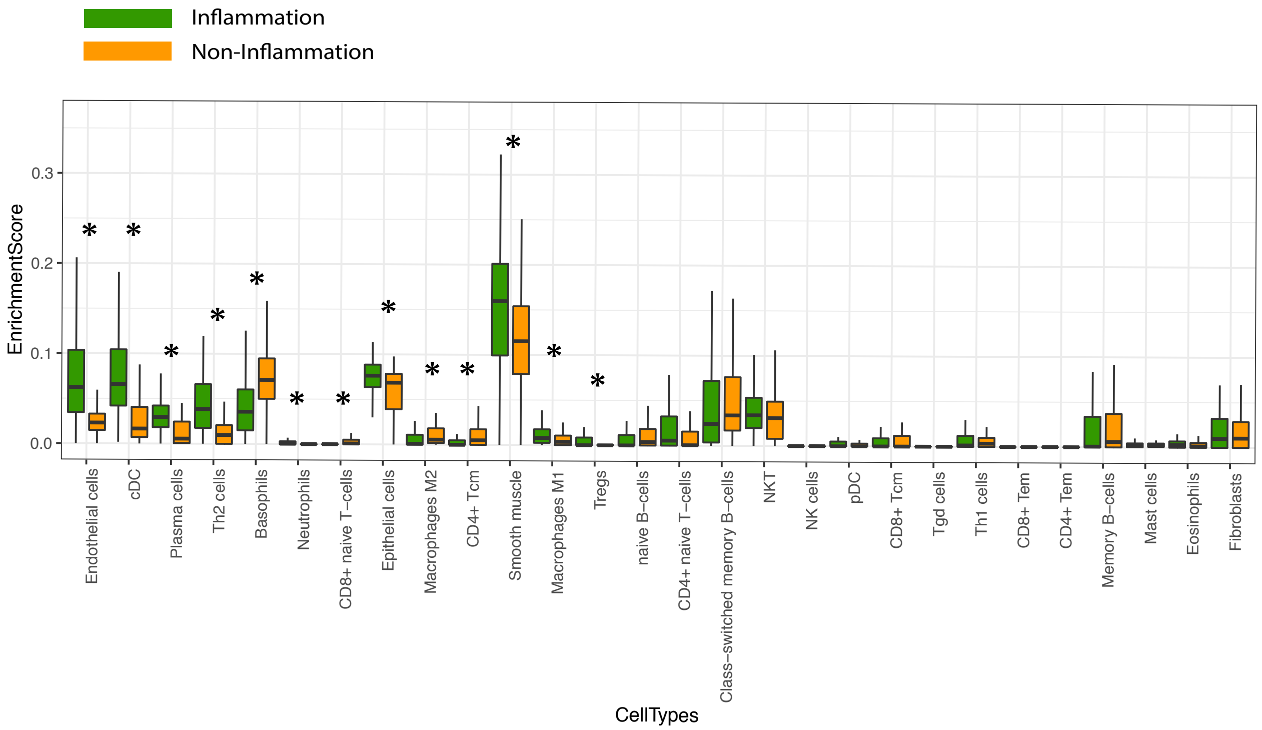

Supplementary Figure 2. Replication of *cis*-eQTLs in GTEx, 'CEDAR' and the eQTLGen study. A) Replication rate (99.15 %) between *cis*-eQTLs identified in this study and terminal ileum *cis*-eQTLs in GTEx. B) Replication rate (97.48%) between *cis*-eQTLs identified in this study and sigmoid *cis*-eQTLs in GTEx. C) Replication rate (99.27%) between *cis*-eQTLs identified in this study and transverse colon *cis*-eQTLs in GTEx. D) Replication rate (92.86%) between *cis*-eQTLs identified in this study and ileum cis-eQTLs in the 'CEDAR' study. E) Replication rate (92.25%) between *cis*-eQTLs identified in this study and transverse cis-eQTLs in the 'CEDAR' study. F) Replication rate (94.64%) between *cis*-eQTLs identified in this study and rectum cis-eQTLs in the 'CEDAR' study G) Replication rate (81.44%) between *cis*-eQTLs identified in this study and blood *cis*-eQTLs in the eQTLGen study (Source data are provided as a Source Data file).

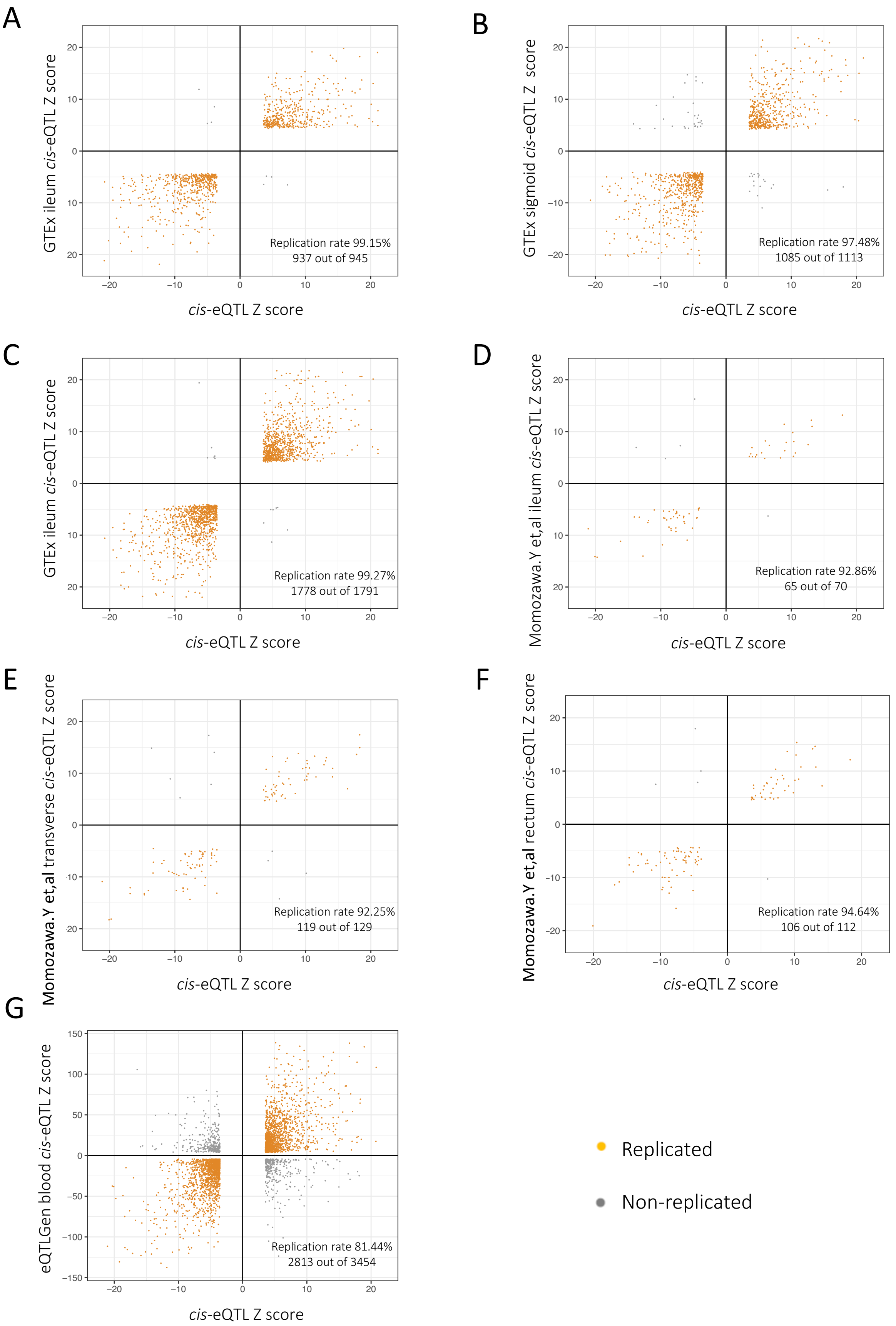
A
GTEx ileum cis-eQTL Z score
cis-eQTL Z score
Replication rate 99.15%
937 out of 945
B
GTEx sigmoid cis-eQTL Z score
cis-eQTL Z score
Replication rate 97.48%
1085 out of 1113
C
GTEx ileum cis-eQTL Z score
cis-eQTL Z score
Replication rate 99.27%
1778 out of 1791
D
Momozawa.Y et,al ileum cis-eQTL Z score
cis-eQTL Z score
Replication rate 92.86%
65 out of 70
E
Momozawa.Y et,al transverse cis-eQTL Z score
cis-eQTL Z score
Replication rate 92.25%
119 out of 129
F
Momozawa.Y et,al rectum cis-eQTL Z score
cis-eQTL Z score
Replication rate 94.64%
106 out of 112
G
eQTLGen blood cis-eQTL Z score
cis-eQTL Z score
Replication rate 81.44%
2813 out of 3454
Replicated
Non-replicated

Supplementary Figure 3. Three potentially IBD-dependent cis-eQTLs. A) *cis*-eQTL effect between SNP rs4342945 and gene *PPP2R2D* (T/C, linear regression, *t*-test, beta =-0.14, FDR =0.00059), B) *cis*-eQTL effect between SNP rs10748 and gene *RBL2* (T/C, linear regression, *t*-test, beta =0.19, FDR =7.29e-07), C) *cis*-eQTL effect between SNP rs2742414 and gene *LIMD1* (T/C, linear regression, *t*-test, beta =-0.14, FDR =0.04). The left panels are *cis*-eQTLs derived from this study while the right panels are derived from GTEx (v8) (https://gtexportal.org/home/). X axis indicates the genotype of eSNP and Y axis indicates the scaled expression level of eGenes. *Cis*-eQTL effect between rs8768 and gene *ZNF593* was not found in GTEx v8. Box plots show medians and the first and third quartiles (the 25th and 75th percentiles), respectively. The upper and lower whiskers extend the largest and smallest value no further than 1.5*IQR (n =280 samples, source data are provided as a Source Data file).

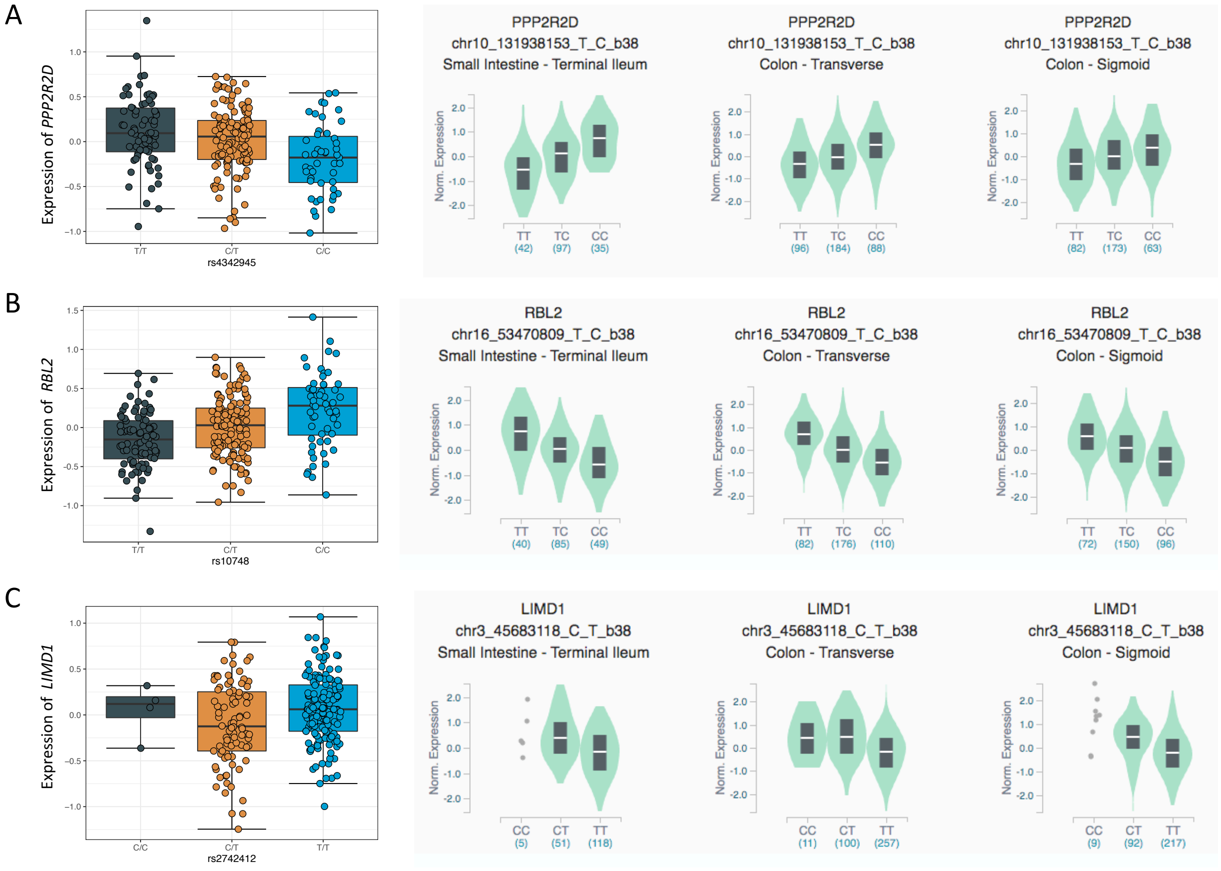

Supplementary Figure 4. Permutation test for inflammation-dependent *cis*-eQTLs. 10x permutations were done for inflammation-dependent *cis*-eQTL analysis. On average, 49 random significant (linear regression, *t*-test, $FDR_{interaction}$ <0.05, red line) *cis*-eQTLs were identified using permutations. In the actual non-permutation analysis, 1,854 significant (linear regression, *t*-test, $FDR_{interaction}$ <0.05) *cis*-eQTLs were identified, which suggests an FDR of ~2.67% (Source data are provided as a Source Data file).

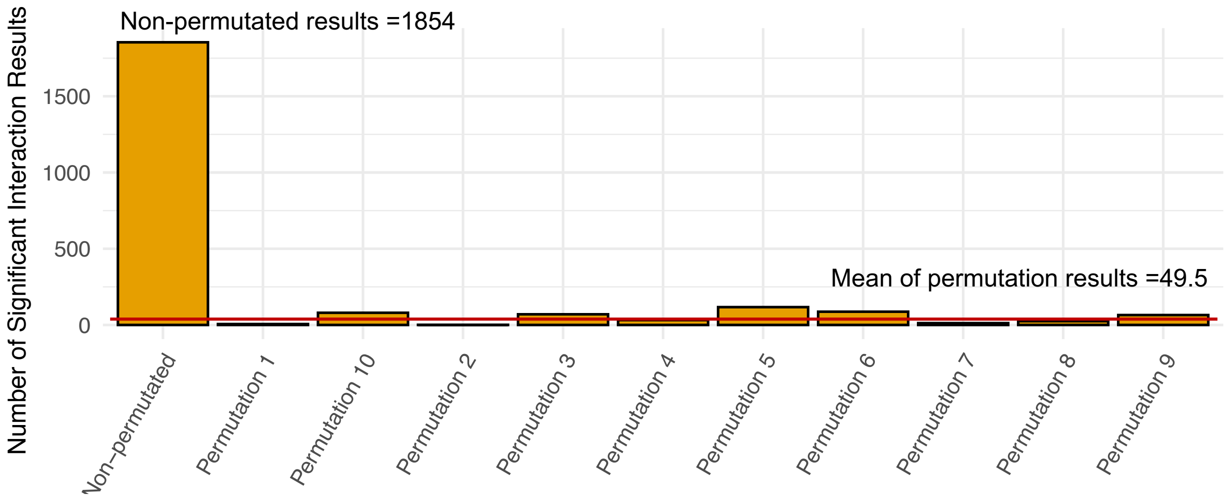

Supplementary Figure 5. Gene expression variation explained by principal components (PCs). X axis indicates the number of PCs. Y axis indicates the total explained variance. Red line indicates the first 18 PCs, which together correspond to 77% of the explained variance (Source data are provided as a Source Data file).

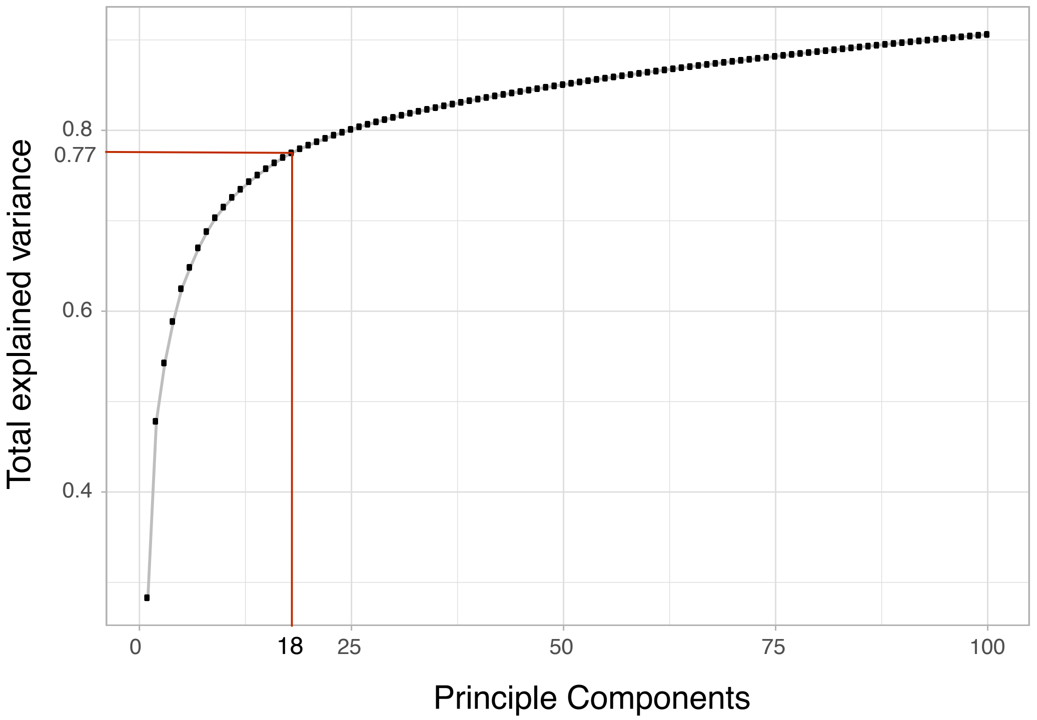

Supplementary Figure 6. PCs and known factors contributing to the variation in gene expression. A) The first two PCs explain ~48% of gene expression variation. Each dot indicates one biopsy sample. The first PC is relevant to biopsy location and disease subphenotype (right and left panel). The second PC is relevant to inflammation status (middle panel). B) Associations between the first PCs and known factors. Barplot, gene expression variance explained by each of first 18 PCs. Heatmap, each square indicates the correlation (R^2, Spearman correlation) between the PC and known factors that could potentially cause differential gene expression (Source data are provided as a Source Data file).

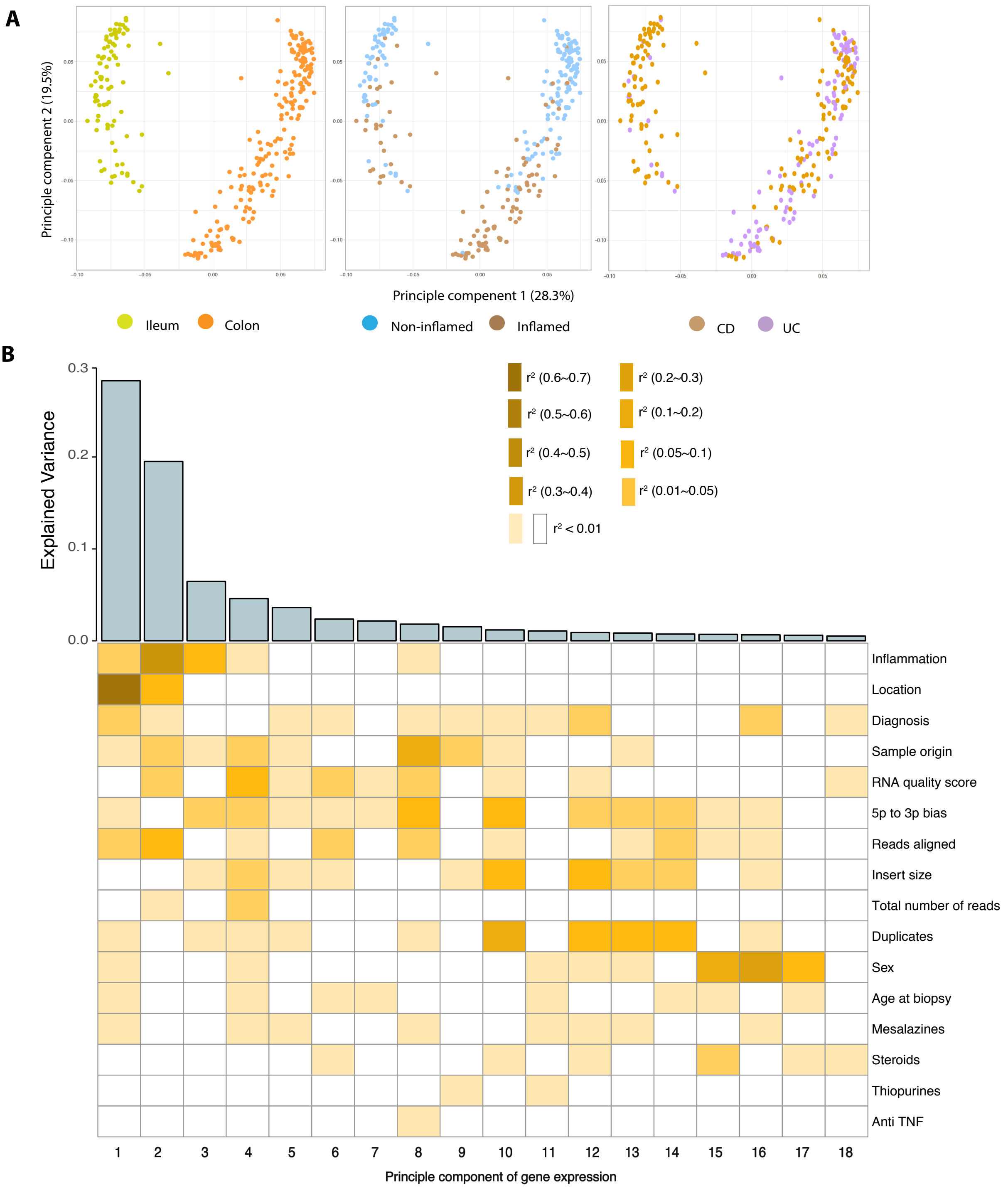
A
Principle compenent 2 (19.5%)
Principle compenent 1 (28.3%)
Ileum
Colon
Non-inflamed
Inflamed
CD
UC
B
Explained Variance
0.3
0.2
0.1
0.0
r² (0.6~0.7)
r² (0.5~0.6)
r² (0.4~0.5)
r² (0.3~0.4)
r² (0.2~0.3)
r² (0.1~0.2)
r² (0.05~0.1)
r² (0.01~0.05)
r² < 0.01
Inflammation
Location
Diagnosis
Sample origin
RNA quality score
5p to 3p bias
Reads aligned
Insert size
Total number of reads
Duplicates
Sex
Age at biopsy
Mesalazines
Steroids
Thiopurines
Anti TNF
1 2 3 4 5 6 7 8 9 10 11 12 13 14 15 16 17 18
Principle component of gene expression
