# Supplementary material for: Inflammation status modulates the effect of host genetic variation on intestinal gene expression in inflammatory bowel disease
Source: Nat Commun. 2021 Feb 18;12:1122. doi: 10.1038/s41467-021-21458-z (PMC7892863; doi:10.1038/s41467-021-21458-z)
Supplement: Supplementary file 3 — Descriptions of Additional Supplementary Files [file 41467_2021_21458_MOESM3_ESM.pdf]

Descriptions of Additional Supplementary Files

Supplementary Data 1

Description: Supplementary Data 1a Differentially expressed genes between inflamed biopsies and non-inflamed biopsies (linear regression, two-sided t-test, FDR < 0.001, |beta| > 1.5); Supplementary Data 1b, REACTOME top annotations for genes differentially expressed between inflamed and non-inflamed tissues (P values were obtained from two-sided Wilcoxon test).

Supplementary Data 2

Description: Supplementary Data 2a Differentially expressed genes between CD and UC (linear regression, two-sided t-test, FDR < 0.001, |beta| > 1.5); Supplementary Data 2b, REACTOME top annotations for genes differentially expressed between CD and UC (P values were obtained from two-sided Wilcoxon test).

Supplementary Data 3

Description:

Supplementary Data 3a Differentially expressed genes between colon and ileum (linear regression, two-sided t-test, FDR < 0.001, |beta| > 1.5); Supplementary Data 3b, REACTOME top annotations for genes differentially expressed between colon and ileum (P values were obtained from two-sided Wilcoxon test).

Supplementary Data 4

Description: Supplementary Data 4a 8,881 unique *cis*-eQTL pairs (linear regression, two-sided t-test, FDR <0.05); Supplementary Data 4b Replication of 39 out of 47 RISK *cis*-eQTLs (Marigorta, UM, et, al (2017). Nature Genetics, 49 (10), 1517.); Supplementary Data 4c Q test of six eQTLs in this study with different directions of effect compared with three GTEx datasets (Cochran-Q test).

Supplementary Data 5

Description: Supplementary Data 5a *cis*-eQTLs colocalized with IBD GWAS (De Lange, KM, et, al (2017). Nature Genetics, 49 (2), 256-261.); Supplementary Data 5b *cis*-eQTLs colocalized with CD GWAS (De Lange, KM, et, al (2017). Nature Genetics, 49 (2), 256-261.); Supplementary Data 5c *cis*-eQTLs colocalized with UC GWAS (De Lange, KM, et, al (2017). Nature Genetics, 49 (2), 256-261.); Supplementary Data 5d *cis*-eQTLs colocalized with colon cancer GWAS (ukb-b-16890); Supplementary Data 5e *cis*-eQTLs colocalized with diverticulitis GWAS (ukb-b-14796); Supplementary Data 5f *cis*-eQTLs colocalized with coeliac GWAS (ukb-b-8631).

Supplementary Data 6

Description: Supplementary Data 6a 190 inflammation-dependent *cis*-eQTLs after multiple testing correction (linear regression, two-sided t-test, FDR < 0.05).; Supplementary Data 6b Full summary statistics for 190 inflammation-dependent *cis*-eQTLs after multiple testing correction (linear regression, two-sided t-test, FDR < 0.05).

Supplementary Data 7

Description: REACTOME annotations of 190 inflammation-dependent *cis*-eQTLs (P values were obtained from two-sided Wilcoxon test).

Supplementary Data 8

Description: 2,466 co-expressed genes amongst 190 inflammation-dependent eGenes (Spearman correlation, |*r*| >0.5, FDR < 0.05)

Supplementary Data 9

Supplementary Data 9 125 inflammation-dependent *cis*-eQTLs interacting with celltype enrichment (linear regression, two-sided t-test, FDR interaction <0.05).

Supplementary Data 10

Supplementary Data 10a Annotation regulatory features of intestinal eQTLs at Haploreg; Supplementary Data 10b Annotation regulatory features of inflammation-dependent eQTLs at Haploreg (P values were obtained from Chi-square tests).

Supplementary Data 11

Supplementary Data 11 Drug Targets overlapping with Inflammation-dependent eQTLs (sources: Open Targets and DrugBank).
