# Supplementary material for: Inflammation status modulates the effect of host genetic variation on intestinal gene expression in inflammatory bowel disease
Source: Nat Commun. 2021 Feb 18;12:1122. doi: 10.1038/s41467-021-21458-z (PMC7892863; doi:10.1038/s41467-021-21458-z)
Supplement: Supplementary file 15 — Reporting Summary [file 41467_2021_21458_MOESM15_ESM.pdf]

nature research

Corresponding author(s): Prof. Dr. R.K.Weersma

Last updated by author(s): Dec 7, 2020

Reporting Summary

Nature Research wishes to improve the reproducibility of the work that we publish. This form provides structure for consistency and transparency in reporting. For further information on Nature Research policies, see our Editorial Policies and the Editorial Policy Checklist.

Statistics

For all statistical analyses, confirm that the following items are present in the figure legend, table legend, main text, or Methods section.

n/a	Confirmed	
☐	☒	The exact sample size (n) for each experimental group/condition, given as a discrete number and unit of measurement
☐	☒	A statement on whether measurements were taken from distinct samples or whether the same sample was measured repeatedly
☐	☒	The statistical test(s) used AND whether they are one- or two-sided<br>*Only common tests should be described solely by name; describe more complex techniques in the Methods section.*
☐	☒	A description of all covariates tested
☐	☒	A description of any assumptions or corrections, such as tests of normality and adjustment for multiple comparisons
☐	☒	A full description of the statistical parameters including central tendency (e.g. means) or other basic estimates (e.g. regression coefficient) AND variation (e.g. standard deviation) or associated estimates of uncertainty (e.g. confidence intervals)
☐	☒	For null hypothesis testing, the test statistic (e.g. F, t, r) with confidence intervals, effect sizes, degrees of freedom and P value noted<br>*Give P values as exact values whenever suitable.*
☒	☐	For Bayesian analysis, information on the choice of priors and Markov chain Monte Carlo settings
☒	☐	For hierarchical and complex designs, identification of the appropriate level for tests and full reporting of outcomes
☐	☒	Estimates of effect sizes (e.g. Cohen's d, Pearson's r), indicating how they were calculated

Our web collection on statistics for biologists contains articles on many of the points above.

Software and code

Policy information about availability of computer code

Data collection	No software was used for data collection
Data analysis	PLINK 1.9; FastQC (v0.11.7); Cutadapt (v1.1); Sickle (v1.200); HISAT (v0.1.6); SAMtools (v0.1.19); Picard tools (v2.9.0); HTSeq (0.9.1); GEMMA (0.98.1); GeneNetwork (https://genenetwork.nl/); Haploreg v4.1;<br>R (v.3.5.0);<br>Codes used for the following data processing and analysis are publicly available at: [https://github.com/WeersmaLabIBD/RNA-SEQ] (DOI: 10.5281/zenodo.4304528)

For manuscripts utilizing custom algorithms or software that are central to the research but not yet described in published literature, software must be made available to editors and reviewers. We strongly encourage code deposition in a community repository (e.g. GitHub). See the Nature Research guidelines for submitting code & software for further information.

Data

Policy information about availability of data

All manuscripts must include a data availability statement. This statement should provide the following information, where applicable:

- Accession codes, unique identifiers, or web links for publicly available datasets
- A list of figures that have associated raw data
- A description of any restrictions on data availability

The raw gene expression table and full eQTL summaries data generated in this study have been deposited in the Genome-phenome Archive data repository database under accession code EGAS00001002702 ("Multi-omics data of 1000 Inflammatory Bowel Disease patients", https://www.ebi.ac.uk/ega/studies/EGAS00001002702, datasets number: EGAD00001006789, EGAD00001006790, EGAD00001006791, EGAD00001006792, EGAD00001006798). Source data are provided with this paper. Due to participant confidentiality, the raw sequencing data and clinical phenotype data are available upon request to the University

Medical Center of Groningen (UMCG), through the submission of a letter of intent to the 1000 IBD Data Access Committee UMCG. The publicly-available datasets used in this study includes the following without any re-processing: six diseases GWAS summary statistics were downloaded from https://www.ebi.ac.uk/gwas/, including IBD (ebi-a-GCST004131), CD (ebi-a-GCST004132), UC (ebi-a-GCST004133), coeliac disease (ukb-b-8631), diverticulitis (ukb-b-14796) and colon cancer (ukb-b-20145); GTEx significant cis-eQTL summary statistics were from (https://gtexportal.org/home/datasets, GTEx_Analysis_v7_eQTL.tar.gz); the 'CEDAR' study intestinal eQTLs were from https://www.nature.com/articles/s41467-018-04365-8; eQTLGen blood eQTLs are from https://www.eqtlgen.org/; the pediatric IBD 'RISK' cohort eQTLs results are from https://www.nature.com/articles/ng.3936. The remaining data are available within the Article or from the authors upon request.

Field-specific reporting

Please select the one below that is the best fit for your research. If you are not sure, read the appropriate sections before making your selection.

☒ Life sciences ☐ Behavioural & social sciences ☐ Ecological, evolutionary & environmental sciences

For a reference copy of the document with all sections, see nature.com/documents/nr-reporting-summary-flat.pdf

Life sciences study design

All studies must disclose on these points even when the disclosure is negative.

Sample size	Sample size (n =299) was determined upon availability of sample numbers. Proper statistical tests and multiple testing correction was performed.
Data exclusions	Samples with no clearly defined inflammatory/non-inflammatory status and poor data quality were excluded (n =19)
Replication	The cis-eQTL pairs from this study were compared with GTEx project, the replication rate is >97%;<br>compared with 'CEDAR' cohort study, the replication rate is >92%;<br>compared with the pediatric IBD 'RISK' cohort study, the replication rate is 83%;<br>compared with eQTLGen meta-analysis, the replication rate is 81.44%
Randomization	No randomization was done. Covariates including age, sex, BMI, smoking, medication and sequence batch were corrected based on multiple principle components.
Blinding	This is an observational study so no blinding of samples was performed.

Reporting for specific materials, systems and methods

We require information from authors about some types of materials, experimental systems and methods used in many studies. Here, indicate whether each material, system or method listed is relevant to your study. If you are not sure if a list item applies to your research, read the appropriate section before selecting a response.

Materials & experimental systems

n/a	Involved in the study	
☒	☐	Antibodies
☒	☐	Eukaryotic cell lines
☒	☐	Palaeontology and archaeology
☒	☐	Animals and other organisms
☐	☒	Human research participants
☒	☐	Clinical data
☒	☐	Dual use research of concern

Methods

n/a	Involved in the study	
☒	☐	ChIP-seq
☒	☐	Flow cytometry
☒	☐	MRI-based neuroimaging

Human research participants

Policy information about studies involving human research participants

Population characteristics	We included one IBD cohort from University Medical Center Groningen which includes an inflamed biopsy dataset and a non-inflamed biopsy dataset.<br>1) inflamed biopsy dataset: mean age (at biopsy collection) 42.7, SD=15.7, 61% female<br>2) non-inflamed biopsy dataset: mean age (at biopsy collection) 42, SD=15.1, 61% female
Recruitment	Participants volunteer in sample collection. Informed consent forms were available for all participants and all were 18 years or older at the time of sample collection. There is no self-selection standard.
Ethics oversight	All participants signed an informed consent form prior to sample collection. This study was approved by the Medical Ethics Committee of the University Medical Center Groningen (Groningen, the Netherlands, IRB ID 2008.338).

Note that full information on the approval of the study protocol must also be provided in the manuscript.
